# Treatment of idiopathic overactive bladder with botulinum toxin: real-life results and patients’ expectations

**DOI:** 10.1097/j.pbj.0000000000000164

**Published:** 2022-05-18

**Authors:** Pedro Abreu-Mendes, Inês Portugal-Rodrigues, Luis Vale, Paulo Dinis, Francisco Cruz, Tiago Antunes-Lopes, Carlos Martins-Silva

**Affiliations:** a Serviço de Urologia, Centro Hospitalar Universitário de São João; b Faculdade de Medicina, Universidade do Porto, Porto, Portugal.

**Keywords:** intravesical treatment, onabotulinumtoxinA, overactive bladder, urgency urinary incontinence, urinary

## Abstract

**Background:**

Overactive bladder (OAB) is a prevalent syndrome affecting 11% to 16% of the adult population. When first-line pharmacological therapy is not effective, intradetrusorial injections of onabotulinumtoxinA (BTX-A) might have an important role in controlling symptoms. The main aim of this study was to access both the efficacy and safety of intradetrusor injections of 100U BTX-A in real clinical practice, among women with idiopathic OAB (iOAB).

**Methods:**

Retrospective study, based on clinical diaries in 136 iOAB female patients, with or without urinary incontinence, submitted to BTX-A injections, between 2005 and 2018 in a tertiary university hospital. Positive response was considered only when the patient mentioned she had great improvement after the injection, otherwise, it was considered negative.

**Results:**

A positive response was obtained in 90 patients (66%) after the first injection. Women with a positive response after the first treatment had 7.5 times more chances to improve with the second (*P* *=* .01). Discontinuation of the therapy after the first injection was neither dependent on the presence of incontinence at baseline (*P* = .73) nor it was related to age (*P* = .6). On univariate analyses, none of the parameters evaluated was useful of predicting successful response, although there was a trend in women who had had a previous midurethral sling surgery for stress urinary incontinence, to have a lower chance of having a positive response after the first injection (*P* = .06).

Thirty-nine women (29%) had at least 1 adverse event, urinary tract infection, and straining to void were the most frequent. Women above 65 years old had less risk of developing a urinary tract infection (*P* = .04).

**Conclusion:**

In real clinical practice, BTX-A injection is an effective (66%) and safe treatment, capable of improving quality of life. Moreover, responding to the first injection seems to predict good clinical outcomes in the second treatment. This procedure can be done with minimal restrictions.

## Introduction

Overactive bladder (OAB) is a prevalent syndrome affecting 11% to 16% of the adult population and can even be more overwhelming in the elderly.^1^ It has a significant impact on quality of life, particularly in sleep disturbance, mental health- related issues and decreased work productivity.^2^,^3^ OAB is characterized by urgency, the core symptom, with or without urgency urinary incontinence (UI), usually with increased daytime frequency and nocturia.^4^ Although urgency UI may suggest detrusor overactivity, it is not necessary to urodynami-cally demonstrate involuntary contractions of the muscle.^5^ OAB is coined as idiopathic (iOAB) when a specific metabolic or pathologic condition that can be the cause is not identified.^3^

The first-line treatment of OAB includes lifestyle changes and behavioral therapy. When these measures are insufficient, pharmacological prescription is the next step.^2^ The first-line pharmacologic option is monotherapy with antimuscarinics or β3-adrenoceptor agonists, while a combination of drugs from both classes can be effective after its failure.^6^,^7^

In patients refractory or intolerant to antimuscarinics or β3- adrenoceptor agonists, evidence supports the use of intravesical injection of 100 U of onabotulinumtoxinA (BTX-A) as a second-line therapy.^8^,^9^,^10^,^11^,^12^ The injections, in between 10 and 20 sites, are done directly into the detrusor muscle, preventing its contractility modulating both efferent and afferent pathways, resulting in a decrease of frequency and number of incontinence e pisodes.^13^,^14^ This treatment has a success rate ranged from 60% to 80%^15^ and is considered safe, durable, and cost- effective.^11^,^13^,^16^ However, some adverse events (AEs) might happen, such as urinary tract infection (UTI) and urinary retention.^16^,^17^,^18^

Unfortunately, not every patient benefit from this minimally invasive therapeutic option.^19^ Therefore, it is important to understand if there are any clinical or nonclinical parameters predicting a good outcome when offering it.^20^

The main aim of this study was to access the efficacy and safety of intradetrusor injections of 100U BTX-A in real clinical practice. In addition, another objective was to search for predictors of positive response and to identify risk factors associated with AEs, to level-up the selection of patients proposed to BTX-A treatment.

## Materials and methods

This was a retrospective study including 136 iOAB women, with or without UI (OAB wet/DRY) who were treated with intravesical injection of 100 U of BTX-A, between 2005 and 2018, in a tertiary university hospital. All patients were older than 18 years and could have initiated and/or ended the treatment at any point in the abovementioned timeframe. Patients whose OAB could have a potential identifiable cause were excluded: neurologic conditions, metabolic conditions (diabetes mellitus type I or type 2 insulin-treated) or other related pathologic conditions. Data were extracted from the electronic health records.

Demographic and clinical data obtained were age, gynecologic and urologic previous history (recurrent UTIs, OAB wet versus OAB dry, presence of mixed urinary incontinence (MUI), previous midurethral sling (MUS), previous pharmacological treatments, number of intravesical injections performed, treatment benefit according to the patient reports (“greatly improved”, “partially improved, not relevant”, “worsened”, “not changed”), reason to abandon therapy and AE after each procedure. Since treatment response was measured as a dichotomous variable, a positive response was considered only when the patient mentioned “greatly improvement”, otherwise it was considered negative. Age was also considered a dichotomous variable (<65 vs ≥65years).

The interval between BTX-A injections was measured from the date of the last injection until the patient asked for the next one.

All the statistical analyses were performed using IBM SPSS Statistics version 25 (IBM Corp., Armonk, NY). To identify baseline variables associated with the effect of the first BTX-A injection (dichotomous outcome) and the occurrence of AE (dichotomous outcome), logistic regression models were fit for each potential predictor. Initially, each baseline variable was modeled individually to assess the association with each of the 2 outcomes. Subsequently, multivariate backward stepwise regression analysis was performed using all variables from the univariate analysis. For proportional comparisons, a chi-square test was used, unless otherwise stated. Mean and standard deviations were used for reported parametric continuous variables. A *P* value < .05 was considered significant.

## Results

A total of 136 female patients with iOAB refractory or intolerant to oral pharmacological treatment were submitted to an intravesical injection of BTX-A. The mean age at first injection was 60.7 ± 15.3 years (22–87). Globally, 268 treatments were performed and the mean number of injections per patient was 1.96 ± 1.32 (1–8).

OAB Wet was present in 121 (89%) women and MUI was found in 99 (72.8%) patients. Nearly one third (n *=* 33) of women with MUI had previously done an MUS surgery for stress urinary incontinence (SUI). A total of 28 patients (22.1%) had a history of recurrent UTI before the injection (Table 1).

**Table 1:** Demographic characteristics of the patients

Variable	
Age at first injection (yr)	60.7 ± 15.3
Parity	2 ± 1.5
Previous hysterectomy	23 (16.9%)
Recurrent urinary tract infection	28 (22.1%)
OAB wet	121 (89%)
MUI	99 (72.8%)
Previous MUS in MUI	33 (24.3%)

Continuous variables are mean ± standard deviation; categorical variables are n (%). MUI = mixed urinary incontinence, MUS = midurethral sling, OAB = overactive bladder.

Concerning the number of treatments, 66 (48.5%) were submitted to a single treatment (n *=* 66), 28.7% discontinued at the second treatment (n *=* 39), and 11% at the third 3 (n *=* 15). The remaining 11.8% of the patients discontinued after 4 or more injections. All patients could ask for a new BTX-A treatment at the time of reappearance or worsening of OAB symptoms.

The mean timeframe between the first and second treatment was 17.5 ± 11.7 months, and the interval between the second and the third was 19.6 ± 14.8months. No significant different was found between these 2 intervals (*P* *=* .4).

Regarding previous pharmacological therapies, 30.9% (n *=* 42) of the patients had tried exclusively an antimuscarinic drug, whereas 7.4% of them (n *=* 10) had tried exclusively the β3 agonist. Combination therapy with 2 antimuscarinic was tried in 25.7% (n *=* 35), whereas 27.9% (n *=* 38) tried the combination of an antimuscarinic with the β3 agonist.

Likewise, there was no statistically significant difference when analyzing age (<65 or ≥65 years old) of women who went for the first injection (*P* *=* .6). In addition, no difference was found regarding incontinence: OAB wet was present in 61 (44.9%) women older than 65 years and in 59 (43.4%) younger than 65 years, although OAB dry was present in more women younger than 65years (*P* *=* .052). MUI was more common in the elderly group (P *=* .03) (Table 2).

**Table 2:** Characteristics by age groups

Variable	<65 yr	≥65 yr	*P*^∗^
Age at first injection	71	65	.6
	52.2%	47.8%	
OAB dry	12	4	.052
	8.8%	2.9%	
Absence of MUI	27	7	.003
	19.9%	7.4%	
Recurrent UTI	17	13	.5
	12.5%	9.6%	
Discontinuation after the first injection	33	33	.62
	24.3%	24.3%	

MUI = mixed urinary incontinence, OAB = overactive bladder, UTI = urinary tract infection.

### Efficacy

Concerning the treatment efficacy, 90 out of the 136 patients (66.2%) had great clinical improvement, 41 (30.1%) reported a suboptimal improvement or even worsened the complaints, and 5 (3.7%) were not evaluated in any appointment after the procedure, having no clinical records.

Analyzing the 66 patients who were submitted to only one treatment, the reasons given to not repeat the injection were multiple: 14 reported lack of improvement, 4 reported worsening of the SUI symptoms, 2 reported worsening of urgency, and 7 referred partial improvement, not relevant enough to repeat. A total of 27 women (40%) decided not to repeat the injection because they considered their symptoms remained controlled after 1 procedure—either with general measures or pharmacological therapy. Nine women were lost to follow-up, although 4 of them had reported clinical improvement in immediate postoperative evaluation. Three are waiting for a new procedure (Table 3).

**Table 3:** Breakdown of repeated onabotulinumtoxinA injections and reasons for discontinuation

Injections, no	1	2	3	4	5	6	8
Patients	136	70	31	16	6	3	2
Decided not to have further injection Reasons given for no further injection, no	63	39	14	10	3	1	2
Symptoms controlled	27	18	9	5	2	1	1
Worsening of SUI	4	0	0	0	0	0	0
Worsening of OAB	2	1	1	0	0	0	0
Loss of effect over time	0	5	3	5	1	0	1
Not significant improvement	7	0	0	0	0	0	0
LTFU	9	4	0	0	0	0	0
Waiting for a new injection	3	0	1	0	0	0	0

LTFU = lost to follow-up, OAB = overactive bladder, SUI = stress urinary incontinence.

Twenty-seven women reported a sustained improvement of the symptoms, without the need to repeat the procedure, and 55 who improved decided to repeat the procedure due to the loss of the effect over time.

Discontinuation of the therapy at the first injection was neither dependent on the presence of incontinence at baseline (*P* *=* .73) nor it was related to age (*P* *=* .6).

On univariate analyses, several parameters, such as age, presence of OAB wet, MUI, number of deliveries, previous hysterectomy and recurrent ITU, were not useful for prediction of clinical outcome after the first BTX-A injection. Although not statistically significant, there was a trend in women who had had a previous MUS surgery for SUI, to have a lower chance of having a positive response in the first injection (odds ratio *=* 0.45 95%; confidence interval: 0.1951.034; *P* *=* .06). In multivariate analysis, no factor was associated with positive treatment response (Table 4).

**Table 4:** Univariate and multivariate analysis of first injection response according to patient demographics

	Outcome after the first injection OR (95% CI) Univariate analyses	*P*^∗^	OR (95% CI) Multivariate analyses	*P*^∗^
Age ≥65 years	1.51 (0.721–3.18)	.27	–	>.05
Parity	1.25 (0.912–1.714)	.17	–	>.05
Previous hysterectomy	0.7 (0.264–1.838)	.46	–	>.05
Recurrent UTI	0.87 (0.371–2.112)	.78	–	>.05
Presence of MUI	0.9 (0.394–2.065)	.8	–	>.05
Previous MUS in MUI	0.45 (0–195–1.034)	.06	–	>.05
OAB wet	1.1 (0.354–3.486)	.86	–	>.05

CI = confidence interval, MUI = mixed urinary incontinence, MUS = midurethral sling, OAB = overactive bladder, OR = odds ratio, UTI = urinary tract infection.

Of those 70 women who were submitted to a second injection, 42 (60%) reported to feel clinically better, 25 (35.8%) experienced no symptomatic improvement or had even a symptomatic worsening and 3 (4.3%) were lost to follow-up without a postinjection evaluation. Of the 42 patients with symptomatic improvement, a total of 20 discontinued BTX-A treatment: 18 after a sustained positive clinical response, 1 after loss of effect over time, and 1 was lost to follow-up. Globally, 31 patients underwent a third injection: 22 women had improved after the second injection, and 9 of them decided to undergo a third treatment, although their expectations were not met after the second one.

From patients submitted to a second injection (n = 70), 55 had a positive response in the first treatment. Regarding this 55, 13 did not respond in the second treatment or the partial improvement did not meet their expectations and 2 worsened. Nevertheless, 38 reported again a clinical improvement. In conclusion, women who had a positive response after the first treatment had 7.5 times more chances to improve after the second (odds ratio = 7.46 95% confidence interval 2.038–27.337; *P* = .01).

### Safety

The total number of AE was 39 (28.7%), all of them grades I and II according to Clavien-Dindo classification. Considering all the injections given, 39 (28.7%) women had at least 1 AE of any kind, 27 (19.9%) presented a UTI after the procedure, 14 (10.3%) had straining to void and 8 (5.9%) required clean intermittent catheterization (CIC) after operation.

On univariate analyses, considering all the injections given, we tried to identify potential predictors of complications, such as age, presence of OAB wet, previous MUS, and history of recurrent UTI, but no association was found. Addressing postoperative UTI, older age (≥65 years) was the only predictor associated with less risk (OR *=* 0.38; *P* *=* .039). Furthermore, none of the predictors was associated with need for CIC. Multivariate analyses revealed that age was an independent predictor of the occurrence of UTI after the procedure (Table 5).

**Table 5:** Univariate and multivariate analyses of all adverse events and separately according to patient demographics

	AEs		UTI		Straining to void		CIC	
				
	OR (95% CI)	*P*^∗^	OR (95% CI)	*P*^∗^	OR (95% CI)	*P*^∗^	OR (95% CI)	*P*^∗^
Univariate analyses								
Age ≥65 yr	0.5 (0.23–1.08)	.08	0.38 (0.15–9.5)	.039	0.57 (0.18–1.81)	.34	0.14 (0.02–1.19)	.07
Recurrent UTI	0.62 (0.26–1.46)	.27	0.6 (0.23–1.55)	.29	0.68 (0.19–2.33)	.54	0.25 (0.06–1.1)	.065
Previous MUS	1.1 (0.46–2.63)	.84	0.89 (0.34–2.35)	.82	1.1 (0.31–4.57)	.79	0.51 (0.11–2.26)	.38
OAB wet	2.1 (0.73–6.22)	.16	1.4 (0.41–4.76)	.58	3.67 (1.01–13.5)	.05	1.1 (0.12–9.36)	.95
Multivariate analyses								
Age ≥65 yr	–	>.05	0.39 (0.15–0.98)	.04	–	>.05	–	>.05
Recurrent UTI	–	>.05	–	>.05	–	>.05	–	>.05
Previous MUS	–	>.05	–	>.05	–	>.05	–	>.05
OAB Wet	–	>.05	–	>.05	–	>.05	–	>.05

AE = adverse events, CI = confidence interval, CIC = clean intermittent catheterization, MUS = midurethral sling, OAB = overactive bladder, OR = odds ratio, UTI = urinary tract infection.

After the first intravesical injection, 29 (21.3%) patients experienced at least 1 AE. The 2 most prevalent were UTI (n *=* 11; 8.1%) and straining to void (n = 8; 5.9%), which was present with UTI in 3 more women, 4 women (2.9%) needed CIC, and 3 of them had UTI as well.

Of the 70 women who had a second intravesical injection, only 7 had an AE, even though 3 needed CIC. None of these women had previously required it. Of the 31 women who went to the third injection, 4 (12.9%) had an AE.

## Discussion

This study was conducted to evaluate the clinical efficacy and safety of BTX-A therapy in the real clinical practice in a tertiary center, in opposition to a trial-controlled situation.

Some of our results were not in line with data within the literature. In our study, we found that age was not a predictor of treatment response, unlike other authors such as Richter et al^20^ found that the treatment in older women with refractory iOAB had less efficacy compared with younger women. Like in our study, Liao et al^21^ found no difference in treatment response during the first 12 months post-BTX-A, when comparing younger patients and elderly patients without frailty. When analyzing the characteristics of our patients by their age (<65 or >65-year-old), we identified some significant differences in features like the presence of incontinence or MUI (both more frequent in older patients). Despite these important differences, the results were similar in both groups, which lead us to think of BTX-A more like an inclusive treatment.

Miotla et al^22^ showed that the efficacy of BTX-A injection in women who had or had not a previous midurethral sling surgery was comparable, whereas in our study there seems to be a trend to worse clinical outcome in patients with a history of previous sling procedure (*P* *=* .06).

As to the interval between injections, we found that it was longer than the usually reported in the literature, 6- to 9-months’ interval. Maybe, one of the main causes for delaying a second treatment is the fact that in our department the treatment is performed in the operating room under sedation and, some patients tend to avoid such procedure.

Concerning side effects, in our clinical practice bladder, BTX-A injection is well tolerated, with most of the AE being limited to the urinary tract. AE after the BTX-A injection affects 20% to 43% of the patients according to the literature,^23^ which is in line with our results. Our figures might be underrated due to multiple factors: on one hand, AEs are usually mild and amenable to treat by a general practitioner and end up not being reported in the clinical diaries. On the other hand, we believe that well informed patients in the real clinical practice do not report minor AE that they were aware of and resolve spontaneously (eg, abdominal straining). The need for CIC in our patients was in line with other studies (1.3%–42.2%), as well as the prevalence of UTIs reported (1.3%–64%).^24^

In our study, age was not a predictor for any kind of complications, except for UTIs. Despite not being consistent with other studies,^21^,^25^ in our study younger patients had a higher rate of UTIs. This might be due to a less active sexual life in older patients. A more detailed comprehensive study involving sexual activity, and possibly with postvoiding residual evaluation, would be interesting. We believe this shows the generalized application of BTX-A injections as a treatment for OAB regardless of patients’ age, as already mentioned above. Elderly women can be proposed for this treatment, always with the needed precaution.

In our department, it is recommended a postoperative evaluation after 2 weeks to rule out elevated bladder residual volumes. We assume that the cases with the highest PVR, probably higher than 150 to 200 cm^3^, correspond to the 8 patients who needed to perform CIC—a rate below the incidence reported in other studies. The women who needed this procedure after the second and third procedures did not need it before.

In our study, most patients who underwent a second procedure, 55 out of 70, did it because they had a positive response to the first treatment. We also found that age has no influence on requesting to repeat the treatment. In some patients, with a negative response in the first treatment, a second treatment was offered since technical issues could have been the cause of the failure. Patients who responded positively to the first treatment were more prone to respond favorably to a second one (*P* = .01). This could lead us to suspect that a favorable clinical response to the first treatment can be used as a good predictor of a successful response to a second treatment.

The biggest limitation of our study is its retrospective nature and the different type of clinical registers between urologists diaries, namely the absence of a questionnaire/score or bladder diary in the preinjection attendance, making it harder to objectively quantify the patient improvement which makes it likely to have an interviewer bias.^19^ On the other hand, iOAB syndrome treatment results might be very subjective and highly individual, influenced by the patient lifestyle and treatment expectations.^19^ The patients’ perception of the treatment result more important than incontinence scores to decide whether or not advance to another treatment.

Since it is not mandatory to have a pretreatment urodynamic study in every patient with OAB, only a few number of patients made the study or had the result registered in clinical diaries, preventing the evaluation of the treatment effect on urodynamic parameters.

Due to its inherent characteristics, this was an exploratory study more than a confirmatory one. Although we could not identify any clinical predictor of positive response to the first BTX-A injection, we can considerer that the response the response to the first injection can itself be a predictive factor to the response to the second treatment.

## Conclusion

We conclude that in the real clinical practice BTX-A intravesical injection (either a unique or repeated treatment) is an effective (66%) and safe treatment. Moreover, the response to the first injection indicates a predictor of good clinical outcomes in a second treatment.

## Acknowledgments

Assistance with the study: none.

Financial support and sponsorship: none.

## Conflicts of interest

F.C. has been a consultant, speaker, or investigator to the following corporations: Allergan, Astellas, Ipsen, Pfizer, and Recordati. No other authors have disclosures.
